# A Community-Engaged Approach to Creating a Mobile HIV Prevention App for Black Women: Focus Group Study to Determine Preferences via Prototype Demos

**DOI:** 10.2196/18437

**Published:** 2020-07-24

**Authors:** Rasheeta Chandler, Natalie Hernandez, Dominique Guillaume, Shanaika Grandoit, Desiré Branch-Ellis, Marguerita Lightfoot

**Affiliations:** 1 Nell Hodgson Woodruff School of Nursing Emory University Atlanta, GA United States; 2 Department of Community Health and Preventive Medicine Morehouse School of Medicine Atlanta, GA United States; 3 Rollins School of Public Health Emory University Atlanta, GA United States; 4 Center for AIDS Prevention Studies Division of Prevention Science, Department of Medicine University of California San Franciso San Francisco, CA United States

**Keywords:** mHealth app, mobile technology, Black women, HIV prevention, reproductive health, women’s health

## Abstract

**Background:**

Black women are an important but relatively overlooked at-risk group in HIV prevention efforts. Although there is an aggregate decline of HIV diagnoses among women in the United States, there are persistent disparate rates of new HIV infections among Black women compared to any other cisgender female subgroup. Strategies to end the HIV epidemic—as outlined in the Ending the HIV Epidemic initiative—for all communities must consider HIV prevention messaging and message delivery mediums that are created with community input. Although mobile health (mHealth) is a popular platform for delivering HIV interventions, there are currently no mobile apps that consider cisgender Black women with the goal of promoting a comprehensive women’s reproductive health and HIV prevention lifestyle. Previous research recommends inclusion of the target population from project inception and iteratively throughout development, to promote use of the intervention.

**Objective:**

The purpose of this study is to understand cisgender Black women’s preferences for functionality, format, and design of a mobile HIV prevention app and to examine their willingness to use an app for HIV prevention.

**Methods:**

We conducted a series of four focus groups with 23 Black cisgender women. Focus groups included discussion and demonstration elements to address cisgender women’s general preference for apps, HIV prevention content that would be useful in an app, and preferred app features that would promote use of an HIV-centric app. During focus group discussions, participants were shown narrated, custom wireframes of HIV prevention app prototypes to demonstrate potential app function.

**Results:**

Findings indicated the presence of eight subthemes within the coding structure of three overall themes: (1) health content within the mobile app, (2) mobile app functionality, format, and design, and (3) other suggested features. Specifically, participants detailed preferred educational content, content distribution, app aesthetics, privacy considerations, and marketing of the app.

**Conclusions:**

Findings suggest that Black cisgender women preferred an app that integrated HIV prevention and optimal sexual health promotion. Participants provided a range of preferences for content integration and facilitators of app engagement with an HIV prevention app. Preferences centered on gender and cultural congruency of information and content, evidenced by visuals, language, and resources. Black cisgender women are viable consumers for a mobile app–based HIV prevention intervention.

## Introduction

Nearly 40,000 Americans were newly diagnosed with HIV in 2017 [1]. Although there has been an 8% decline in new HIV diagnoses, individuals of color—particularly Black women—continue to be disproportionally affected by the current HIV epidemic, trailing only men who have sex with men (MSM) [2,3]. Black individuals are the most affected racial or ethnic group, with Black men having a lifetime HIV risk of 1 in 20 and Black women having a risk of 1 in 48, compared to 1 in 132 and 1 in 880 for White men and White women, respectively [4]. In 2017, Black women accounted for almost 60% of newly diagnosed HIV infections among women and comprised 26% of HIV incidences among the Black population in the United States [3,5].

Black heterosexual women have a higher rate of HIV and sexually transmitted infections (STIs) compared to any other female group in the United States [1]. There are several social determinants of health that contribute to this increased HIV risk among this population [6]. These include poverty, lack of access to quality health care, unstable housing, sex-based power differentials in couple relationships that limit women's ability to negotiate HIV protective actions with their regular male sex partners, limited HIV prevention education, the increased prevalence of other STIs, and alcohol and drug use [6,7]. In addition to these factors, it is also important to consider that many Black women are at an increased risk for HIV acquisition due the mere concentration of people living with HIV within their geographical area or communities, coupled with their sexual networks [8]. Black women themselves may have few sexual partners; however, they are more likely to have sexual contact with individuals who have multiple sexual partners [8,9]. Thus, Black women may remain at high risk for contracting HIV even when they do not exhibit risky behaviors [8].These factors are important to recognize when addressing HIV prevention interventions for Black women in high-risk areas, such as the southern United States.

Evidence-based, face-to-face behavioral interventions (eg, SiHLE [Sisters, Informing, Healing, Living, Empowering] and Horizons) [10] that involve educational sessions led primarily by Black women have been effective in reducing disparities in HIV transmission [7,11]. Although effective, such interventions have been greatly limited by factors related to cost, scalability, sustainability, and reproducibility [7,12]. For example, barriers commonly associated with traditional interventions are limitations related to ease of access (eg, location of study activities), cost (eg, childcare), transportation difficulties, and time constraints, which can prevent study enrollment and engagement and/or lead to high study attrition [13]. Technologically delivered behavioral interventions driven by mobile health (mHealth) have the ability to overcome such barriers while offering several advantages compared to traditional face-to-face interventions [13]. Promising implications for the use of mobile technology to facilitate the delivery of health care rely on general mobile phone usage by the target population.

Among Black youth and residents of low-income households, smartphone ownership exceeds 80% and plays a critical role in providing internet access, compared to laptops or desktop computers [9,14-16]. As a result, Black youth and adults are more likely than White people to rely on their smartphones for certain activities, including seeking health care information [14]. The increasing popularity of smartphones along with smartphone apps have made the possibility of employing mobile phones and apps as a platform to provide HIV prevention information for Black women highly feasible [17].

HIV prevention interventions delivered through mobile apps have been largely aimed at MSM [18,19]; yet, they have failed to attract the attention of, and positive responses from, other ideal audiences, particularly Black women [18]. Studies that have focused on mHealth for HIV prevention among Black women have primarily utilized telephone calling and text-based counseling interventions, which have demonstrated marked efficacy in reducing risk behaviors and incident STIs among women compared to face-to-face counseling sessions [10,12]. Although interventions that have assessed texting and calling as HIV prevention interventions have been effective, there is a need for the development of mHealth interventions that specifically target Black women and use innovative and resourceful approaches that promote user engagement and sustainability. The few research studies that have taken on this approach have shown promising results. Jones and Lacroix [9] employed an mHealth intervention for Black women that used smartphones to deliver culturally tailored, soap opera videos that focused on HIV prevention for young Black women to encourage a reduction of high-risk behaviors. The video intervention was compared to weekly text-based HIV risk–reduction messages, which tend to be more commonplace in mHealth HIV prevention efforts. The intervention arm showed a dramatic reduction in unprotected sexual encounters postintervention when compared to baseline [9]. Gonzalez Gladstein [20] conducted a pilot randomized controlled trial that evaluated the feasibility and efficacy of a web-based app to increase HIV and STI knowledge and use among Black and Latina young adult women. Study participants in the intervention arm found the app to be trustworthy and useful, and results revealed that participants in the intervention arm had high levels of engagement and retention [20]. Currently, Browne et al [21] is conducting a randomized controlled trial that involves trialing a mobile app to provide HIV prevention and risk-reduction education to young African American women living in North Carolina who are sexually active and use drugs. If efficacious, this mHealth app can be useful in expanding the reach of HIV risk–reduction interventions for Black women and increasing accessibility for Black women who use mobile devices [21].

The etiology of HIV transmission among Black women largely differs from that of other high-risk populations, further emphasizing the need for more tailored interventions to be developed for Black women [7,11]. While HIV prevention efforts targeting MSM are necessary, it is crucial that we engage other vulnerable populations in prevention efforts in order to successfully meet both national and global HIV elimination goals. Black women are not likely to engage with mHealth apps developed for MSM, due to lack of relatability and contextual relevance as it relates to their intersecting HIV prevention and women’s health needs. More specifically, health promotion interventions for Black women focusing on HIV prevention should promote self-empowerment, gender and ethnic pride, self-efficacy, and skills building [7]. Mobile apps developed for Black women have the potential to deliver HIV prevention information and skills in interactive, useful, nonstigmatizing, and discrete ways [7]. Furthermore, Black women are willing to participate in mHealth research that promotes the prevention and management of chronic illnesses, especially when such research is culturally tailored and comprehensively considers their reproductive and sexual health concerns. mHealth purports care continuity and constant accessibility, thus offering users flexibility and convenience with no limitation of time nor space [17,22-26]. In addition, mHealth provides the ability to deliver highly engaging HIV prevention information to populations that have been typically hard to reach, while offering user privacy and anonymity [17]. Considering Black women’s use of mobile apps for the prevention of other chronic diseases, we are optimistic that our proposed HIV prevention mobile app will have similar outcomes [13,27].

This study was guided by the social cognitive theory of mass communication, which postulates four constructs from the original theory—self-efficacy, use of incentive motivation, social environment, and reciprocal determinism—that impact behavior, but adds that messaging to influence these behaviors can be effectively delivered through media and technological sources [28]. In order to understand vulnerable, cisgender, Black women’s preferences for functionality, format, and design of a mobile HIV prevention app, and to examine their willingness to use an app for HIV prevention, we conducted formative qualitative research with Black cisgender women who live in communities that are geographically affirmed to have the highest HIV rates in Atlanta, Georgia [11].

## Methods

### Study Population and Recruitment

This study was approved by the Emory University Institutional Review Board. This qualitative study was implemented from February to March 2019 with cisgender Black women residing in metro Atlanta, specifically Fulton County, one of the targeted counties from the Ending the HIV Epidemic initiative [29]. Participants were recruited for focus group discussions (FGDs) via flyer distribution and community-based organization outreach. Flyers were distributed in venues that Black women frequent, such as beauty salons, churches, community-based organizations, and community events like health fairs. The flyers provided a link to a survey via the online survey tool SurveyMonkey, which assessed participant eligibility. Inclusion criteria for this research study required participants to be (1) English speaking, (2) cisgender female (ie, assigned female sex at birth and identified as female), (3) 18 years of age or older, (4) self-identified as Black, African American, and/or Hispanic or Latina, and (5) sexually active within the previous 3 months during study enrollment. Additionally, it was required that participants owned a smartphone and had never tested positive for HIV per self-report. Eligible women were contacted by phone to participate in a prescheduled FGD.

### Study Procedures

In total, there were 23 participants who were divided among the four in-person FGDs held. We concluded that our sample size was adequate based on data saturation and empirical evidence of sufficiency [30]. Each FGD lasted approximately 90 minutes and took place at a partnering community-based organization or academic institution. FGDs were conducted by two trained moderators who were knowledgeable about the objectives of the HIV prevention mobile app. We employed an FGD guide (see Multimedia Appendix 1) during each session, and both moderators were present during all sessions to ensure the specific aims of the study were met and to ensure that there was consistency across the data collection procedures. The FGDs covered four main topics: (1) HIV prevention app usability, (2) features of an HIV prevention app, (3) app content to include barriers and facilitators to HIV testing and pre-exposure prophylaxis (PrEP) initiation, and (4) mobile app commodity ordering (eg, condoms and at-home HIV testing kits). During the FGD, we demonstrated four HIV prevention–focused mobile app wireframes—digital depictions of app content and functionality—that were developed by and for Black women. Specific mobile app functions included the following: (1) information about HIV and women’s reproductive health (eg, via videos), (2) location-based HIV testing and PrEP clinics, (3) use of an in-app calendar for reproductive health (eg, ovulation and sexual acts diary) and HIV-specific notifications (eg, testing reminders), (4) commodity ordering for HIV prevention efforts (eg, condoms), (5) sexual behavior tracking, (6) frequently asked questions repository, (7) prevention navigator and/or provider communication, and (8) community connectivity (eg, peer chat group). Participants were also encouraged to provide suggestions for how to improve app function and recommend additional app features that should be integrated. All interviews were digitally recorded with the consent of each participant. Field notes were drafted in real time by the research assistant and later transcribed and appended to the FGD transcripts. Participants were given US $30 in compensation for their time.

### Data Analysis

All FGD audio files were transcribed verbatim using a professional transcription service. Thematic analysis, combining inductive and deductive approaches, was completed using MAXQDA software, version 18 (VERBI GmbH). A codebook was compiled in close coordination between researchers (RC, NH, SG, and DBE) using existing literature, the research objective and aims, along with themes that emerged during the FGDs. The researchers then evaluated the FGD transcripts to ensure congruency with the extracted themes using MAXQDA software. Following this process, the researchers discussed and compared their findings. Transcribed text and field-note data were then reviewed for overall impressions; finally, a line-by-line review for extraction of significant statements occurred [31].

## Results

### Overview

A total of 23 cisgender Black women participated in the FGDs; they ranged in age from 18 to 45 years, with a mean age of 30 years (SD 8). Demographic content was recorded for 17 of the 23 participants (74%), as 6 participants opted not to complete the demographic form (see Table 1). Most of the participants in the study were not married (12/16, 75%), did complete high school (10/16, 63%), had health insurance (15/17, 88%), and had a regular health care provider (15/17, 88%). Over half of the participants reported they had previously heard of PrEP (12/17, 71%) and less than half reported that they would not use PrEP (7/16, 44%). Broadly, participants reported their main health concerns as chronic diseases, sexual and reproductive health issues, along with inadequate acute and chronic mental health services.

Results were categorized into three overarching themes and eight related subthemes. The first overarching theme, *health content and communication*, included two subthemes: *comprehensive information* and *health provider profiles*. The second theme, *functionality, format, and design of the mobile app*, included three subthemes: *customizability, layout, colors, easy navigation, and simplistic design; safety and privacy concerns;* and *visual content*. The third theme, *other suggested features*, included two subthemes: *peer chat room* *(ie, community building)* and *“tell me where, get me there” transportation*.

**Table 1:** Participant demographics.

Category and description		Value, n (%)^a,b^
**Race (n=17)**		
	Black or African American	16 (94)
	West Indian	1 (6)
**Marital status (n=16)**		
	Married	2 (13)
	Never married or single	12 (75)
	Not married but living with a sexual partner	2 (13)
**Currently in school (n=17)**		
	Yes: full time	6 (35)
	Yes: part time	0 (0)
	No	11 (65)
**Highest level of schooling (n=16)**		
	Elementary or middle school	1 (6)
	High school	10 (63)
	Trade or technical college	1 (6)
	College or university	3 (19)
	Not reported	1 (6)
**Employment status (n=16)**		
	Full time	3 (19)
	Part time	7 (44)
	Unemployed	6 (38)
**Yearly household income (US $; n=16)**		
	0-9999	7 (44)
	10,000-19,999	2 (13)
	20,000-29,999	2 (13)
	30,000-39,999	3 (19)
	40,000-49,999	2 (13)
**Current health insurance (n=17)**		
	Yes	15 (88)
	No	1 (6)
	Unsure	1 (6)
**Access to a regular health care provider (n=17)**		
	Yes	15 (88)
	No	2 (12)
**Have you heard of pre-exposure prophylaxis (PrEP; n=17)**		
	Yes	12 (71)
	No	5 (29)
**If you have heard of PrEP, would you use it (n=16)**		
	Yes	7 (44)
	No	7 (44)
	Maybe	2 (13)
**Where do you obtain health information (n=17)**		
	Family and/or friends	6 (35)
	Google	10 (59)
	Health apps	7 (41)
	Health care provider	15 (88)
	Social media	2 (12)

^a^Out of 23 participants, 6 opted out of completing the demographic document.

^b^Not all category percentages add up to 100 due to rounding.

### Health Content and Communication

#### Overview

The participants expressed the need for a comprehensive health app that included various topics in connection to a woman’s overall health. Along with HIV, participants communicated that the mobile app would be most beneficial and would foster continued use if it included resources on additional health topics that were of concern to them, such as mental health. A directory of providers was a distinct feature desired by participants. More specifically, Black women requested having the app generate health care provider profiles based on geographic location (ie, geofencing) that would locate Black female providers. In regard to communication facilitated by the app, there was a consensus among the participants on having a virtual communication option to access their health care providers using either a face-to-face feature or a private chat room forum. Some participants were interested in having a peer-support option and wanted a monitored peer chat room available. The risk assessment questionnaire that was incorporated in three of four of the mobile app prototypes garnered reactions from participants that should govern how to solicit sensitive information via electronic devices from Black women. Existing app functions were desired; however, participants explicitly expressed wanting all features to be included in one mobile app, thereby minimizing the need to download multiple apps or the need to exit the app (eg, period or mood trackers). Table 2 lists and describes the codes for the themes and subthemes.

**Table 2:** Code definitions.

Code	Definition
Health concerns	Primary health concerns mentioned by participants, including those related to sexual and reproductive health; nutrition, exercise, or weight management; mental health; chronic disease; cancer; and accessibility to care
HIV	Discussions of HIV or HIV-related topics by participants, including HIV prevention methods and strategies they are aware of; experience, utilization, discussion, or knowledge regarding pre-exposure prophylaxis (PrEP); perceptions and preconceived notions regarding HIV; discussions related to HIV, HIV treatment and care, HIV-related illness, and risk factors related to HIV
Information delivery preference	Discussions regarding participants’ preferred methods of receiving health-related information, including those related to the utilization of mobile apps or social media apps to receive health information, seeking health information on the web, and seeking health information from television commercials
Health app features: reliable digital dialogue	Discussions regarding participant preferences in app features and usability, including the following preferences: receiving health information through the app, interacting with health care providers through the app, interacting with other women in an app chat room, and personalized health information received by participants through the risk assessment
Comprehensive app content	Discussions of the app that include comprehensive information pertaining to all aspects of women’s health, discussions about participant ability to search the app for information by typing in specific symptoms, and ability to access provider profiles that include information regarding their services
Convenient consumerism	Discussions regarding participant ability to order health kits, tests, etc, directly from the app
Customizability	Discussions regarding customizing the app and the display of features, including, but not limited to, color, font, layout, background, and music
Visual content	Discussions regarding nontraditional delivery methods of health care information through videos, short clips, and representative imagery
“Tell me where, get me there” transportation	Discussions of transportation and accessibility of services presented in the app, including health care providers within the participant’s geographic area, links and accessibility to Lyft and Uber, and information about community carpools to health care services
Other suggested features	Discussions regarding additional suggestions voiced by participants to ensure efficiency and overall satisfaction with the app (ie, iOS and Android compatibility, journal component, and panic button for emergencies)
Privacy and confidentiality	Discussions regarding security measures to ensure participant confidentiality while using the app

#### Comprehensive Information

Topics that participants voiced wanting to see included in the mobile app consisted of the following: location of health screening services for women (eg, mammograms), mental health coping strategies and local resources, information on chronic health conditions that are most prevalent within the Black community (eg, diabetes and hypertension), holistic wellness, and information on sexual and reproductive health that appends to HIV prevention, such as other STIs.

My three concerns are STDs [sexually transmitted diseases], mental health, and chronic illnesses like high blood pressure, diabetes, and heart issues.

Focus group #3 participant

My three [health concerns] are, well I’d say asthma, I haven’t got a sexually transmitted disease, but I’m just saying it for other women. So, I would say sexually transmitted diseases and I’d say anxiety too.

Focus group #2 participant

**Figure 1:**
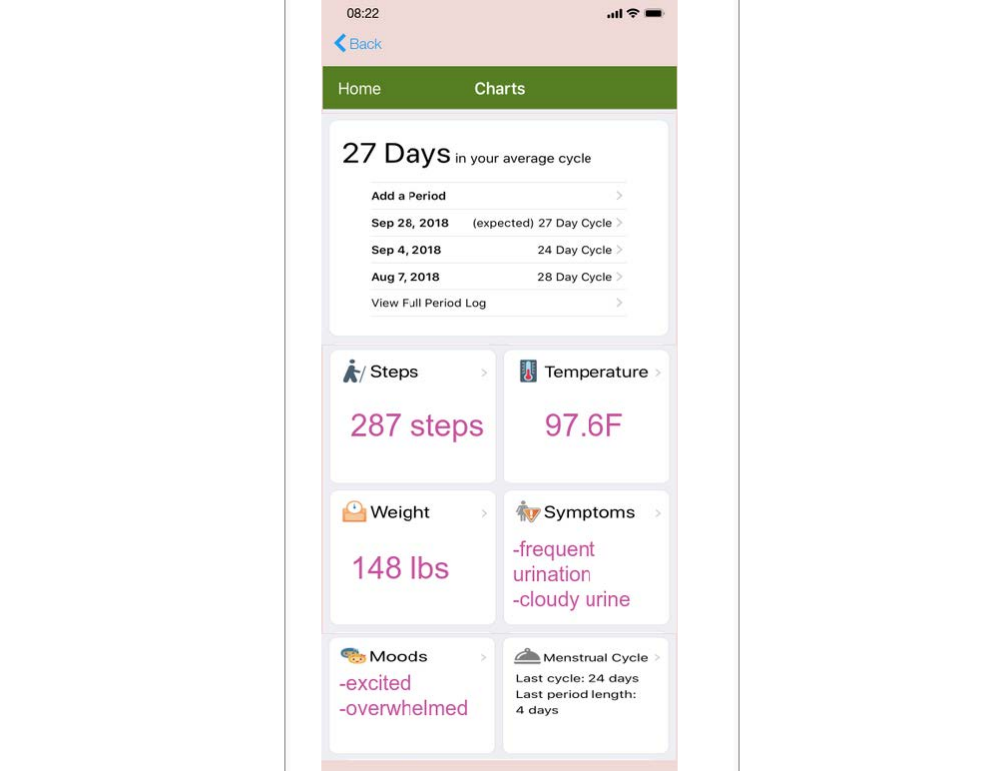
Comprehensive health features included in the sample mobile app prototype.

Participants also expressed an interest in a search engine function. With this feature, women could receive quick, reliable, and relevant information pertaining to common reproductive health trepidations, such as STIs and birth control methods. Additionally, participants recommended the inclusion of a period tracker feature within the app. Several participants already had period tracker apps on their phones and stated that it would be most convenient for them if the app afforded period initiation, duration, and symptom monitoring so that they could refer to just one app for all of their reproductive health needs (see Figure 1). Participants also liked the option of having a journal feature within the app to log journal entries and notes that are relevant to their health.

I like the multipurpose features because if I'm not necessarily looking for HIV information, if it's a well-rounded site and I'm able to get the period tracker, count my steps, and the other features like the news section it's well-rounded for me.

Focus group #2 participant

I think if there's a way to search quick things like yeast infections. A quick way to search things you know about common female health issues. So, you can just go ahead and see what you need to do. Instead of jumping through hoops for something basic you can get out of the way quickly.

Focus group #3 participant

#### Health Providers’ Profiles

**Figure 2:**
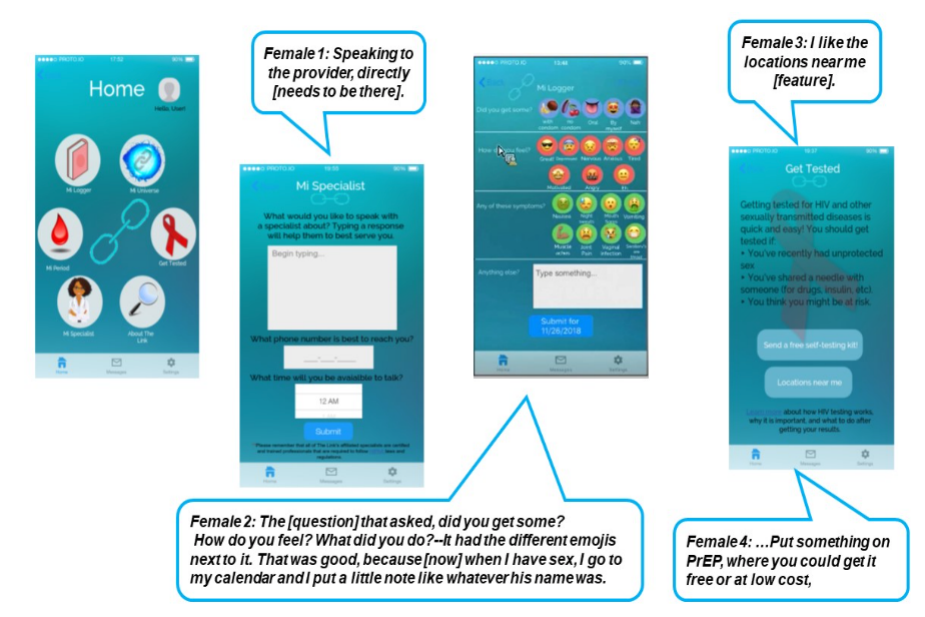
Sample mobile app prototype features and pages. PrEP: pre-exposure prophylaxis.

The participants reported an interest in having the mobile app accommodate a comprehensive directory of female health care providers of color within their communities. For the participants in this study, having access to health care providers they could relate to—considering intersections like race and ethnicity as well as gender—was influential when seeking reproductive and general health services. Participants also preferred having the option of directly communicating with a certified health care provider or a trained health care surrogate (eg, patient navigator) via either in-app video conference or chat room in case they have any immediate health concerns or questions (see Figure 2).

...Or even something where you could literally get a directory of female doctors in your area. Female Black doctors in your area. I think that would be very, very helpful because I have no idea of any Black women doctors in Atlanta. That would be very helpful. [Focus group #2 participant]

A risk assessment that would collect health information about participants was a proposed component of most mobile app prototypes that were presented to the focus group participants. Components of the risk assessment were as follows: sexual encounters, use of contraception, and demographic information, such as age, race, and gender. The participants noted that the placement of the risk assessment was crucial for the overall acceptability of the app. There were differences in opinions among FGD participants as to where the risk assessment should be placed, along with the content of the risk assessment. Some participants stated that having the risk assessment appear immediately after signing in was a deterrent to using the app because the questions being asked were perceived as intimate or invasive. One participant stated that the risk assessment included too many questions, which caused her to become disengaged with the app. Multiple participants expressed that if the risk assessment were to be included in the app, then it should be placed in a different section entirely and serve a purpose (eg, usefulness to the health care provider for giving feedback to the patient regarding a particular health concern) for collecting the type of information being queried. However, there were also some participants, specifically younger participants, who did not have a problem with the placement of the risk assessment nor the content of the questions asked in the risk assessment.

It was for me [risk assessment], right off the bat, I was like, ya know...I was over it. But now what I will say, I think that those questions can be asked, but you would probably do it when they get to certain levels [within the app].

Focus group #1 participant

I feel like even the health tracker thing on your iPhone tells you to put in your weight and your height. I know my BMI is 39, but I also know I'm active as heck. So, yes, you can tell me this but my lifestyle says something different. I feel like I would expect a health app to ask me that. I feel like that would be part of the sign-in process. But I guess I'd maybe give the option to skip [the risk assessment].

Focus group #3 participant

### Functionality, Format, and Design of the Mobile App

#### Overview

Participants were adamant about helping to create a mobile app that would not only be functional but also highly competitive with, and more culturally centric than, all other pre-existing mobile apps concerning reproductive health. These suggestions ranged from design features, layout, navigation within the mobile app, and concerns regarding safety and privacy to visual content and representation.

#### Customizability, Layout, Colors, Easy Navigation, and Simplistic Design

Across all focus groups, the participants discussed the various customization elements they wanted to see incorporated into the mobile app; for example, compatibility with both iOS and Android systems, limited ads within the app, volume options, and notification alerts. They also stressed that the app should be user friendly. This included a color layout with simplistic designs. One participant did note that she would like to see a reproductive app for women that was not “pink and flowery.” Participants also expressed how they liked that the pictures, which were of Black women, within the app felt relatable to them. There was not a clear consensus regarding the use of emojis in the app; as some mentioned, it would be appealing to younger women, while others noted that it would come off as less mature.

I feel like just stay away from the color pink. I feel like I'm very tired of seeing that color associated with women.

Focus group #3 participant

I think a lot of the ads come from funding. I want to talk about my health! If the ads pop up, make it relevant to what you’re looking for. Just no random ads...

Focus group #4 participant

#### Safety and Privacy Concerns

When using technology, privacy is oftentimes one of the major concerns for an individual, and the same could be said of our participants. Many were concerned that their private medical records and HIV status could be inadvertently disclosed. With web-based and smartphone apps allowing multiple ways to sign up and sign in, participants were concerned with their personal information being shared. The majority of participants emphasized not wanting to sign in to the app using social media platforms, such as Facebook or Instagram, due to the fear of their information potentially becoming compromised. If personal health information was required in order to sign up for the app, many participants expressed that they would be less inclined to complete the form due to privacy concerns. Participants also suggested that they would prefer a name for the app that was discrete and did not imply that the app pertained to HIV.

With so much stuff that’s going on with these different social [media] sites, that’s why a lot of people don’t [share information online using these platforms] because they fear that this stuff can get linked back.

Focus group #2 participant

I would rather not do it with anything social media. Especially anything owned by the company Facebook, which includes Instagram. Just because they track and keep everything.

Focus group #3 participant

#### Visual Content

**Figure 3:**
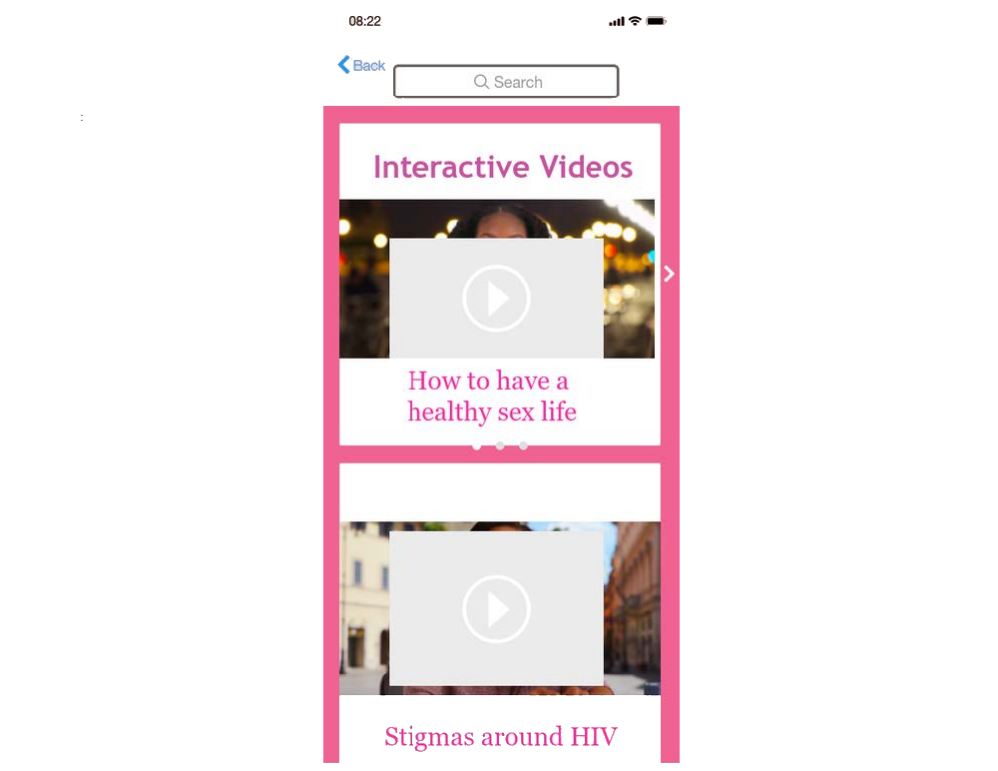
Interactive videos on the mobile app featuring Black women.

Participants were especially interested in receiving their health care information in new, innovative ways. Some participants described including health education resources in the form of short video clips of real-life scenarios (see Figure 3). Others discussed the incorporation of animations throughout the app as a way of providing relevant information. These alternatives to traditional methods of health information delivery were presented as a means of reaching all potential users. Participants suggested that given that literacy levels may vary among users, along with young adults primarily obtaining news and information through videos and clips, health education in the form of videos may be easier for some to understand.

I think in this day and age, people are visual people because of social media. A visual, I was thinking something more like real-life scenarios, whether that be a 60-second video, but something that’s real, something that’s honest, a real story.

Focus group #1 participant

### Other Suggested Features

#### Overview

In addition to the themes and subthemes related to the app health content, functionality, format, and design, participants also provided additional suggested features they would like to see present in the app. This included a peer chat room and information regarding transportation and location of services. Regular news updates on important health topics were also suggested, along with the incorporation of a panic button for emergency situations.

#### Peer Chat Room

There was no clear consensus among the participants regarding a peer chat room. On one hand, some participants were intrigued by this feature, as it would allow an opportunity for community building and an opportunity to learn from others who may be experiencing similar circumstances. Much of the opposition regarding the chat room was related to the potential for sharing of misinformation relating to HIV and STI risks. In addition, participants were concerned with potential misuse of the chat room. They suggested security and privacy measures should be considered, as individuals may be sharing personal information relating to their reproductive health. Some participants preferred having chat rooms solely with health care providers as opposed to the peer chat rooms.

It [the peer chat room] just depends on how compelling it would be. Also, if it's regarding health, I would like a doctor in the chat room.

Focus group #3 participant

...The fact that they’re having these chat rooms and avatars is too...childish. That would push me away because if we’re talking about something serious, I don’t want to make an avatar of myself and I don’t want to be in a chat room talking to a whole bunch of people that may potentially have something that I’m concerned about. Because to me it tends to turn into something more fun than something more serious.

Focus group #2 participant

#### “Tell Me Where, Get Me There” Transportation

Participants stated that a GPS locator for HIV testing and PrEP clinics was a feature they would be interested in seeing incorporated within the app. For some of the participants, although they had heard of PrEP, they did not know where to obtain it. Thus, they stated that having this information accessible would make the process of utilizing such services much easier. Some participants even mentioned partnering with transportation services, such as Uber and Lyft, in order to provide transportation to promote accessibility of health care facilities.

...if [you] could put something on PrEP, where to get it free or at a low cost, because that’s the question I get most of the time, because PrEP is very expensive.

Focus group #2 participant

## Discussion

### Overview

Aims of app development and definitions of success may differ between researchers and programmers, and each may be responding to different perceived needs of end users. Teams can address some of these differences through continuous formative research with the target population during intervention development and creating very explicit deliverables, timelines, and division of labor within the development team [32]. These first and second points emphasize the importance of designing technology-based interventions with the end user constantly in mind to create something that is intuitive, useful, engaging, and fun [32].

### Black Women and the Use of Mobile Technology for Sexual and Reproductive Health

In this study, researchers solicited feedback from focus groups consisting of Black women residing in a high-risk area for HIV acquisition, in order to obtain qualitative data for the development of an HIV prevention mobile app that specifically targets Black women. Black women remain disparate in HIV acquisition and in other adverse sexual and reproductive health outcomes. Features of an HIV prevention and sexual and reproductive mobile health app informed by Black women could help combat health outcome inequalities. The findings obtained from this study indicate that, overall, participants strongly desire a mobile app that incorporates comprehensive health information that discusses HIV prevention information. Participants also strongly desire a mobile app that incorporates information pertaining to other aspects of women’s sexual and reproductive health and information on other health conditions that have a high prevalence within the Black community. One particular aspect of the app that was highly regarded by study participants was the ability for participants to be linked with health care providers within their geographical area who were Black women. Representation oftentimes can play a major role in an individual's health decision making. Research has demonstrated that when Black patients have a health care provider of the same racial background, there tends to be a more patient-positive effect involving patient-centered communication, along with increased satisfaction [33,34]. Thus, by having access to Black providers, Black women may become more engaged in their overall health care, which can facilitate access to more health promotion initiatives, including HIV prevention efforts. Other desired app features may currently exist in some form implicating feasibility, either in related apps (eg, HIV prevention apps for MSM) or unrelated apps (eg, period tracker), but distinction will be demonstrated by culturally and contextually relevant content that Black women will deem useful regarding their sexual and reproductive health. Black women who are skeptical about health resources or who cannot always decipher health content will have a reliable source that will share evidence-based content in a manner that considers their literacy level and preference for information delivery. We also confer that the app will have to be malleable and inclusive as it relates to health issues that disproportionately impact Black women in order to remain relevant and be sustainable. Our participants were enthusiastic about being partners in mobile app development efforts and ensuring that the app was an authentic reflection of their needs.

HIV prevention delivered through mHealth that is specifically developed for Black women can offer a promising strategy to curtail HIV incidence rates within this group.

The data obtained from focus groups will be used to adjust and modify the mobile app we developed to ensure that the app includes the needs and concerns of Black women and to ensure that the app is culturally tailored to the population of interest. Cultural tailoring is a crucial component of HIV prevention interventions, and research has demonstrated that Black youth are more likely to reduce high-risk behaviors and increase condom use when they are able to identify with, and find meaning behind, the education that is provided [7,35,36]. Solely possessing HIV or STI prevention information has shown to not effectively reduce high-risk sexual behaviors [22]; however, greater message frequency and individually tailored messages have been effective in sustaining new health promotion behaviors and reducing high attrition rates [10,37].

### Aptitude When Considering Prior Work

Black women continue to be disproportionately affected by HIV, and there is a great need for strategic interventions that are targeted, scalable, and sustainable in order to help reduce HIV rates among this group [7]. Through mHealth, HIV prevention efforts can be specifically targeted toward the needs of Black women, and they can also be scalable so that more participants are reached compared to more traditional HIV prevention interventions. Compared to traditional interventions, such as group counseling, mHealth reduces the amount of resources needing to be expended, making the sustainability of mHealth more feasible [7]. Although research has demonstrated that Black women are willing to utilize culturally tailored mHealth interventions, there has been a dearth of research assessing mHealth interventions—specifically the use of mobile apps—as a means of providing HIV prevention and risk-reduction education for Black women [25]. Studies that have assessed mHealth as a means of HIV prevention among Black women have focused primarily on web-based and telephone-based (eg, calling or texting) interventions [10,22]. In our review of the literature, we did not identify any articles that speak to the use of mobile apps as a means of HIV prevention for Black women. Although calling and texting interventions have proven to be effective, with the increase in smartphone ownership and mobile app use among Black women, a mobile app will offer a more expansive strategy that relies minimally on human resources for the delivery of health promotion content for this target population. Traditional HIV risk-reduction interventions targeting Black women have demonstrated marked challenges, including maintaining sustained intervention effects over time, along with the feasibility of expanding such interventions to reach more participants [7]. Thus, there is ample potential for the utilization of mobile apps in order to overcome challenges in the delivery of HIV prevention content for Black women.

### Mobile App Considerations

We are not oblivious to the fact that mobile apps have to be maintained and will require some resources for sustainability. We propose integrating this app into an existing health care system, whose administrators can assume ownership of protecting and storing the data, mobile app updates, and tracking patient usage. Our efforts would be to provide a general app that can be assumed by health care agencies (eg, Healthy Start) that deal primarily with female patients of color who oftentimes are not afforded digital services [38]. A recent systematic review revealed the limited representation of Black people and African Americans in health intervention research, even though they experience the greatest burden of health inequities [39]. Additionally, the review highlighted the increased willingness by Black women to participate in mHealth studies [39-41]. It is crucial for HIV prevention interventions delivered through mHealth to expand beyond recurrent themes and groups, such as MSM and medication adherence [40]. Other key populations such as Black women, along with themes such as HIV prevention and care initiation, need to be of focus in mHealth interventions [40]. In developing an mHealth app for Black women, it is important for the app to be revered as essential in order to incentivize Black women to keep it on their phones and permit the app to occupy valuable data space. We deduced that the health concerns of Black women will motivate their use of the mobile app if they can receive some of the same health benefits that are ordinarily afforded to them through traditional engagement with a health care provider or the health care system. There are commercial apps, such as Maven [42], that are available but are not within the purview of our population—the most susceptible to poor reproductive health outcomes—because they cannot afford the services. We, however, plan to integrate the mobile app into an existing health care system that will afford our target population an opportunity to engage with this mobile technology and with their health care providers.

### Limitations

We identified some limitations to this study. One of these included the fact that our sample represented Black women residing in a large metropolitan community. Therefore, the results of this study can only be generalized to women of a similar demographic. Additionally, of the four focus groups, only one targeted the ideal age group for the proposed mobile app, due to recruitment methods and interest in the research project.

### Conclusions

This research project sought to understand the interest in an HIV prevention app by Black women of reproductive age. In eliciting the opinions of Black women through focus groups, the information obtained can be used to ensure that the app that is developed will be relevant to the concerns and needs of Black women. In doing so, we can ensure that the app we develop is both culturally and contextually relevant so that users can be heavily engaged with the goal of promoting positive behavior change and risk reduction. Moving forward, the researchers would like to work alongside an advisory committee of Black women, a research team, and a technology company in order to develop the app prototype.

## Appendix

Multimedia Appendix 1 Focus group discussion guide.

## Abbreviations

FGD: focus group discussion
mHealth: mobile health
MSM: men who have sex with men
PrEP: pre-exposure prophylaxis
SiHLE: Sisters, Informing, Healing, Living, Empowering
STI: sexually transmitted infection
