# Supplementary material for: A Community-Engaged Approach to Creating a Mobile HIV Prevention App for Black Women: Focus Group Study to Determine Preferences via Prototype Demos
Source: JMIR Mhealth Uhealth. 2020 Jul 24;8(7):e18437. doi: 10.2196/18437 (PMC7414400; doi:10.2196/18437)
Supplement: Multimedia Appendix 1 [file mhealth_v8i7e18437_app1.docx]

*Mobile HIV Prevention App for Black Women: Preferences and Prototype*

**Focus Group Guide**

**Introduction:**

Thank you for coming to talk with us today. We appreciate you taking time from your day to help us learn more about keeping young women your age healthy.

My name is XXXX and this is (name of Co-Investigator; Research Assistant [RA]). We will be working together to help the group go smoothly.


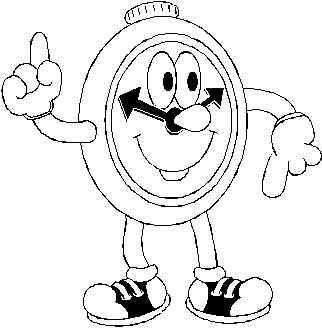
This meeting will last around **1 hour and 30 minutes**. We have some drinks and snacks here for you. Bathrooms are located….


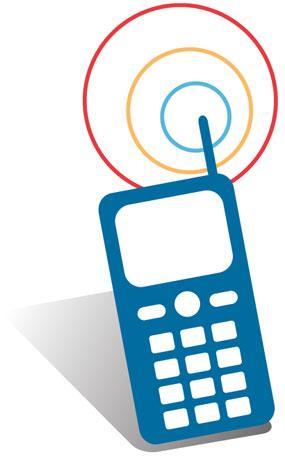
*We ask that you please turn off your cell phones so you don’t get interrupted while we’re talking. If you can’t turn off your phone for safety reasons, please put it on vibrate only.*

**Group Guidelines:**

Before we start, we need to **agree** on some guidelines to make sure that everyone feels comfortable.

We’ll be talking about **private** information in this group, and it’s important that you all feel that what you share will not be shared with others. So, please don’t share what’s been said here with people who are not in the group.

It’s important that we show **respect** for each other in the group. We will sometimes disagree with each other and that’s normal. We’ll ask that you listen to others, even if you disagree. We will also want to hear your views. Also, please try not to speak when someone else is talking. We want to hear from everyone in the group: you all have something important to add.

We’ll ask that you be as **open and honest** as you can in the group. We can learn from each other, and everyone has something valuable to share. Also keep your mind open to other points of views. There are no right or wrong answers to these questions.


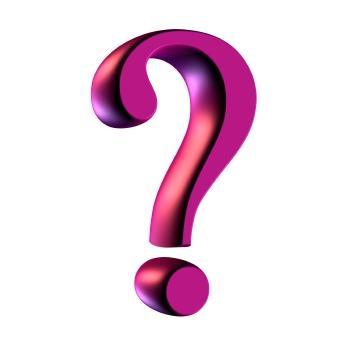
*You may ask questions. We will try to answer any questions when they are asked, but we may need to wait until the end of group if the answer is complicated or doesn’t have to do with the goals of the group.*

*
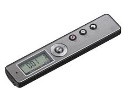
 All group sessions will be digitally recorded.*


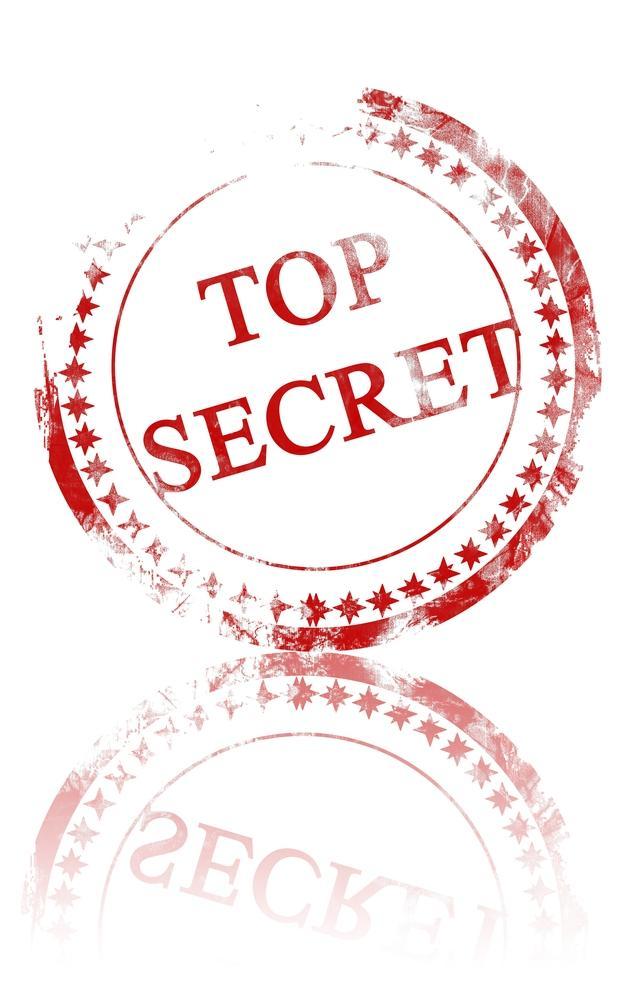
*Your privacy and confidentiality will be protected at all times. Only the research team, including the transcribers, which are people who translate the audio tapings into a written document, will listen to the recordings.*

*Participants may choose to use their first name only or an alias (alternative) name. Please do not use last names of yourselves or others (including people in your lives). Do you have questions about the taping?*

**Can we all agree to these guidelines?**


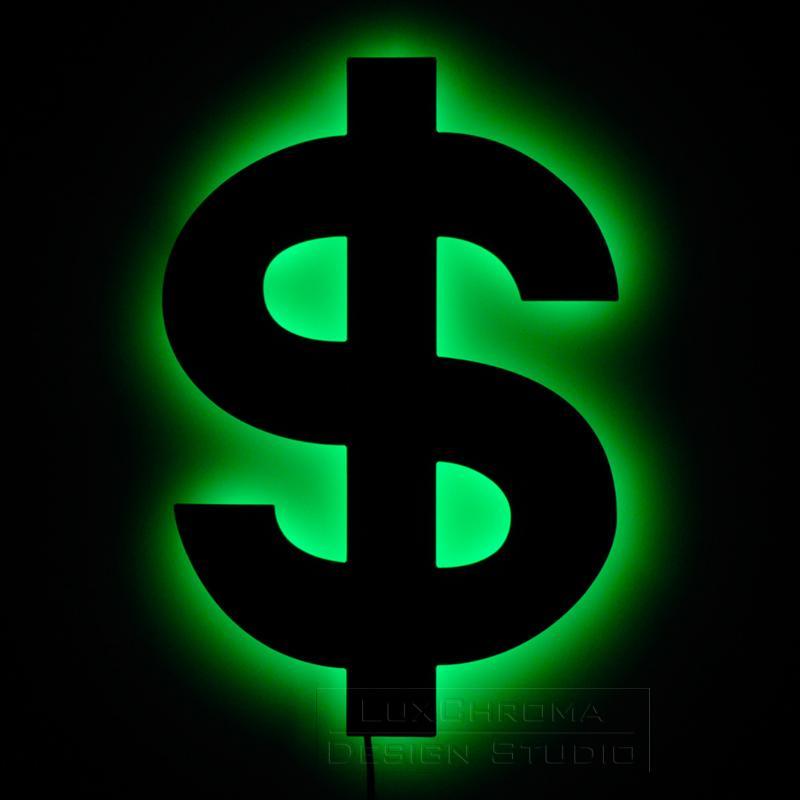
 Because we appreciate you taking the time to help us learn more about what young women your age think, and because we understand you may have expenses as a result of attending, we will be paying each of you $30.00 for being a part of this group. **PAYMENT** will be provided at the end of the session and each of you will need to sign a receipt.

**Preliminary Question:**

Let’s go around the group and introduce ourselves. Please use a first name or alias only.

My name is Rasheeta Chandler and I work at the Emory University, School of Nursing as a researcher & instructor. Now I will let (RA name here) introduce themselves.

As we mentioned earlier, we will be working together to help the group go smoothly. (RA name here) will also be taking notes so we don’t miss any important information.

**PURPOSE AND GOALS:**

**There are two main goals for tonight’s group:**

**1) To learn what all of you think about having an/a HIV prevention/Reproductive health promotion mobile application focused on the needs of Black women; and 2) To learn what mobile app features would encourage continued use of the mobile app.**

*Some of the things that we’re going to be talking about, like sex, can be embarrassing or difficult to talk about. We understand this, and we will help each other to be more comfortable. One more thing about talking about sex—it is very important for group members to feel that their behavior or choices are not being judged. There is a wide range of sexual behaviors. We're all responsible for making our own choices; in this group, we will also be accepting of others' choices. You have the opportunity to learn a lot from each other - as long as members feel comfortable sharing.*

**
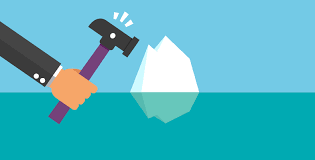
 Icebreaker:** What is you alias/first name and what song describes you today?

**General health Questions:**

- Name for me your top three health concerns?

**Transition:**

**Safe Sex/HIV prevention Information Needs of Black Women: [5 Questions]**

What type of information do you think women like you need to protect them from acquiring STIs/HIV/ or experiencing an unintended Pregnancy?

- Who do you think is most at risk for HIV?
- What have you heard about Pre-Exposure Prophylaxis (PrEP)?
- Who do you think PrEP is for?
- What have you seen or heard about PrEP in media messages?

**HIV prevention information delivery preference(s) per Black women:**

**Transition:**

*That discussion was very helpful. It sounds like there are some real challenges facing young women who want to practice safe sex or reduce their risk of acquiring HIV. When talking with young women, we have found that some young women feel that certain things in their lives has an impact on their decision to have sex/safe sex.*

**Introductory questions: [13 questions]**

- How would you like to hear information about health topics important to Black women, including HIV?
- If you were to receive health related information on your phone, how would you prefer to receive it— e.g. through a mobile app, search the web, through a social media option?

**Now, we’re going to discuss the needs of you and your friends regarding health applications (apps).**

Why would you start using a health app?

Why would you keeping using a health app for a long period of time?

o Can you provide examples of health apps that you have used for a few weeks or a month?

o Can you provide examples of health apps that you have used for 3 months or longer

- What mobile apps do you use most on your smart phone?
- What are three apps that you continue to use since you’ve had a smart phone, and you continue to download even when you change devices?
- What features in that app do you remember most (e.g., the song/jingle, the acting)?
- What features encourage you to use mobile applications on your cell phone?

What concerns may you have about using health apps?

Prototype Demo [13 Questions]

*Directions: I am now going to ask you look and listen to a few sample mobile apps that were created by Spelman students, to see what you think about them. We want you to pay close attention to the app features you like and must have & those you can do without; then I will ask you a few questions after each demo.*

**Start Prototype demos: Content with Black women’s reproductive health concerns with an emphasis on HIV prevention--A Mobile app for Black Women by Black women**

**Note: Play prototype demo videos. After each video, the following questions will be asked.**

- In this app, what information was most memorable?
- What information in this app should we keep, why?
- What information in this app should be removed, why?
- How easy does the app seem to use or figure out?
  - Is the app easy to navigate?
- Would you pay for this app? [-with your time?]
- Does that app look cool, interesting? Is it eye-catching?

At the end of viewing the prototypes, have participants will summarize which features were most appealing, & which were unappealing; also ask what (if any) specific content was not included that should have been.

- **Out of the features that were included in the app, which did you prefer? Why?**
- **Would you use this app? Why or Why not?**
- **What would keep you using this app?**

**Transition: Recruitment & Retention**

*We really appreciate all the information you have given us. Thank you very much. We have one final area that we would like to talk about. If we were to do a study with Black women focused on sex practices and preventing unintended pregnancies using technology as well as teaching them information about preventing STIs/HIV (Recruitment/Retention):*

- What would be good ways to tell young women about the study?
- What would be a good logos or labels?—Any current logos or labels that you can compare to (What would need to be included in the logo to get your attention?) (Have some options that can be selected)
  - Options will be provided
- What should we call it?

**Final Questions:**

- Is there anything that we didn’t talk about today that you think it would be important for use to know? Did we miss anything?

If you would like the results of the study, let myself or (RA name here) know, when you get your incentive.

**Closing:**

Thank you very much for coming and talking with us today.
